# GATS tag system is compatible with biotin labelling methods for protein analysis

**DOI:** 10.1038/s41598-023-36858-y

**Published:** 2023-06-23

**Authors:** Kohdai Yamada, Fumiya Soga, Soh Tokunaga, Hikaru Nagaoka, Tatsuhiko Ozawa, Hiroyuki Kishi, Eizo Takashima, Tatsuya Sawasaki

**Affiliations:** 1 Division of Cell-Free Life Science, Proteo-Science Center, Ehime University, 3 Bunkyo-Cho, Matsuyama, Ehime 790-8577 Japan; 2 Division of Malaria Research, Proteo-Science Center, 3 Bunkyo-Cho, Matsuyama, Ehime 790-8577 Japan; 3 Department of Immunology, Faculty of Medicine, Academic Assembly, Advanced Antibody Drug Development Center, University of Toyama, Toyama, 930-0194 Japan

**Keywords:** Immunoblotting, Biochemical assays

## Abstract

Polypeptide tags and biotin labelling technologies are widely used for protein analyses in biochemistry and cell biology. However, many peptide tag epitopes contain lysine residues (or amino acids) that are masked after biotinylation. Here, we propose the GATS tag system without a lysine residue and with high sensitivity and low non-specific binding using a rabbit monoclonal antibody against *Plasmodium falciparum* glycosylphosphatidylinositol (GPI)-anchored micronemal antigen (PfGAMA). From 14 monoclonal clones, an Ra3 clone was selected as it recognized an epitope—TLSVGVQNTF—without a lysine residue; this antibody and epitope tag set was called the GATS tag system. Surface plasmon resonance analysis showed that the tag system had a high affinity of 8.71 × 10^–9^ M. GATS tag indicated a very low background with remarkably high sensitivity and specificity in immunoblotting using the lysates of mammalian cells. It also showed a high sensitivity for immunoprecipitation and immunostaining of cultured human cells. The tag system was highly sensitive in both biotin labelling methods for proteins using NHS-Sulfo-biotin and BioID (proximity-dependent biotin identification) in the human cells, as opposed to a commercially available tag system having lysine residues, which showed reduced sensitivity. These results showed that the GATS tag system is suitable for methods such as BioID involving labelling lysine residues.

## Introduction

Biotin labelling technology has been widely used in many studies, in the life sciences and chemistry. In particular, because N-Hydroxysuccinimide (NHS)-ester can react with the amino groups of proteins, such as lysine residue or N-terminal, NHS-biotin is available in commercial kits and has been used for biotin labelling of proteins. In addition, the BioID (proximity-dependent biotin identification) method has been widely used for protein–protein interaction (PPI) analysis. BioID technology uses proximity biotinylation enzymes such as BioID^1,2^, TurboID^3^, and AirID^4^. The enzyme is fused to the protein of interest (POI) and subsequently carries out biotin labelling of the proximity proteins. These features have been used to perform comprehensive PPI in cells^5^ and organisms^6^. Recently, we developed the proximity biotinylation enzyme AirID^4^ and analyzed the drug-dependent PPI^7^. The BioID enzyme produces biotinyl-5′-AMP as an intermediate^2^, which reacts with the amino group of a side chain in the lysine residue. Therefore, lysine residues in proteins are modified by biotin.

Polypeptide tag technology is used in many PPI analyses, such as co-immunoprecipitation^8^, AlphaScreen assay^9–11^, and protein array^12–14^. Tag systems are also used in many life science experiments, such as cell biology and transgenic organisms. In most life science studies, commercially available peptide tag systems, such as FLAG^15^, MYC^16^, and HA^17^, have been used to detect or analyze target proteins. Surprisingly, many tag systems, except for the HA tag, have a lysine residue in the epitope amino acid. Because the biotin labelling technology described above modifies lysine residues, many tag systems are not suitable for the functional analysis of proteins using biotin labelling. Therefore, a new tag system that excludes lysine residues is required for protein analysis using biotin labelling.

In this study, we developed the GATS tag as a novel tag system that uses a rabbit monoclonal antibody against *Plasmodium falciparum* glycosylphosphatidylinositol (GPI)- anchored micronemal antigen (PfGAMA) localized to malarial micronemes (https://plasmodb.org/plasmo/app/record/gene/PF3D7_0828800). The GATS tag system showed a high affinity of 8.71 × 10^–9^ M on surface plasmon resonance (SPR) and provided high sensitivity and low background on immunoblotting using the lysates of mammalian cells. It has also been used for immunoprecipitation and immunostaining of cultured human cells. Furthermore, the system showed high performance in the detection of proteins in the NHS-biotin and BioID methods, whereas the FLAG tag reduced the sensitivity. This GATS tag system provides a useful tool for the functional analysis of biotin labelling proteins, such as in the BioID method.

## Results

### Isolation and characterization of rabbit monoclonal antibodies against *Plasmodium falciparum* GAMA protein

**Figure 1:**
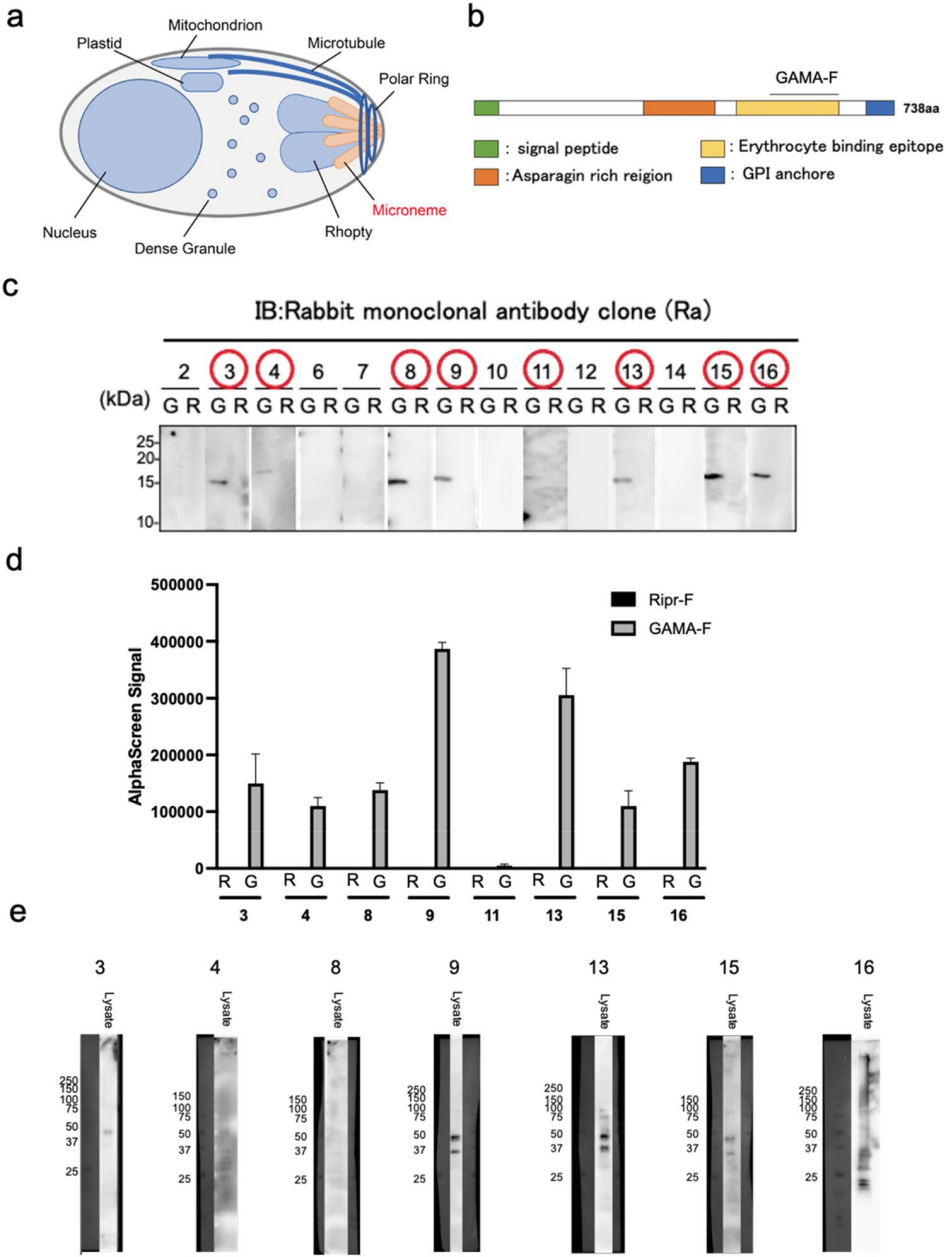
Identification of anti-GAMA rabbit monoclonal antibodies. (**a**) A simplified cellular model of *Plasmodium falciparum*. (**b**) Domain information on the GAMA protein and the location of GAMA-F. (**c**) Image showing immunoblotting evaluation of the binding of antibodies to GAMA-F and Ripr-F (control) synthesized using a wheat cell-free protein synthesis system. Antibodies are unpurified supernatants produced in Expi293F cells. (**d**) In vitro interaction assay of rabbit monoclonal antibodies clone (Ra) and GAMA-F. Ripr-F is used as control. Antibodies are unpurified supernatants produced in Expi293F cells. All AlphaScreen signals are raw luminescent signals in the AlphaScreen-based biochemical assay. Error bars denote the standard deviation (independent experiments; n = 3). (**e**) Evaluation of antibody specificity with lysates of *P. falciparum*. Antibodies are produced in Expi293F cells and purified by protein G Sepharose 4 Fast Flow (GE Healthcare).

GAMA is a protein of the malaria parasite *P. falciparum* (Fig. 1a); it is known to localize on secretory organelles called micronemes^18,19^. We selected the 602aa-715aa in PfGAMA protein because it showed high protein productivity, [GAMA_602-715_, (GAMA-F) Fig. 1b]. The recombinant GAMA-F protein was synthesized as a C-terminal Strep-tag fusion form by a wheat germ cell-free protein synthesis system^20^. The malaria protein PfRipr fragment [Ripr_720-934_,(Ripr-F)]^21^ was used as a control for specificity evaluation (https://plasmodb.org/plasmo/app/record/gene/PF3D7_0323400). Fourteen antibody gene sets consisting of heavy and light chain genes were cloned using the immunospot array assay on a chip (ISAAC) method^21^, and each antibody was expressed in Expi293F cells. Specificity was evaluated by immunoblotting using the antigen GAMA-F (~ 15 kDa) (Fig. 1c). Ripr-F, a part of *P. falciparum* Rh5 interacting protein (PfRipr)^21^ was used as a control for specificity evaluation. Positive antibodies (red circles in Fig. 1c) were used in the binding assay using the AlphaScreen assay. Seven out of eight rabbits (Ra) monoclonal antibody (mAb) clones specifically recognized GAMA-F (Fig. 1d). Furthermore, three (Ra3, Ra9, and Ra13) mAbs showed a clear band in immunoblotting using lysates from *P. falciparum* (blood stage) (Fig. 1e). Therefore, Ra3, Ra9, and Ra13 mAbs were further analyzed.

### Rough epitope mappings of three anti- GAMA-F antibodies

**Figure 2:**
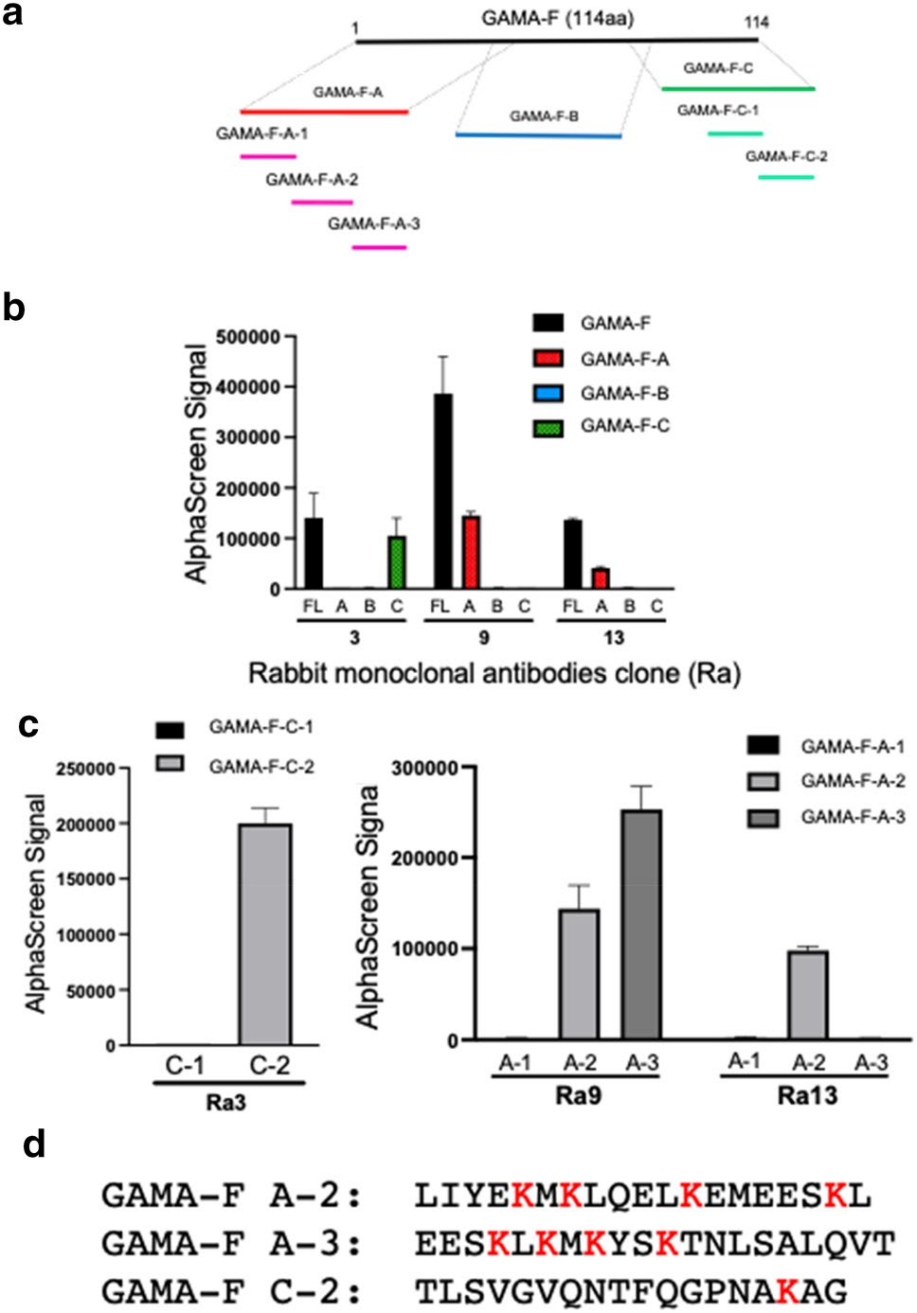
Broad epitope determination of Ra3, Ra9, and Ra13. (**a**) Diagram representing the GAMA-F fragment for epitope determination. (**b**) Identification of epitope region using A, B, and C GAMA-F fragments. These fragments were fused to the N-terminus of GST-TEV-bls protein and synthesized using a wheat germ cell-free system. Binding between the fusion protein and Ra3, Ra9, and Ra13 antibodies was detected by AlphaScreen. (**c**) Identification of epitope region using smaller segmented fragments of A, B, and C. (**d**) Diagram representing the lysine residues of the GAMA-F fragment.

To determine the approximate epitope of Ra mAbs, GAMA-F was divided into three major fragments. The three fragments were named A, B, and C, and were further fragmented to narrow down the epitope in detail (Fig. 2a). All the fragments were synthesized by the wheat cell-free system as a fusion to the C-terminus of GST-TEV-bls and were then used to determine the binding regions of the three antibodies by AlphaScreen (Fig. 2b). Ra3 recognized the GAMA-F-C fragment, and both Ra9 and Ra13 mAbs recognized the GAMA-F-A fragment. Further analysis indicated that Ra3 mAb bound to GAMA-F-C-2, Ra9 mAb bound to both GAMA-F-A-1 and -A-2, and Ra13 mAb bound to GAMA-F-A-2 alone (Fig. 2c). The epitope sequences of these mAbs were determined from these mappings (Fig. 2d). Interestingly, the rough epitope sequence of the Ra3 mAb showed only one lysine residue, whereas the remaining epitope sequence included four lysine residues. Therefore, the Ra3 mAb was used for further analysis.

### Determination of the minimal amino acid sequence recognized by Ra3 mAb

**Figure 3:**
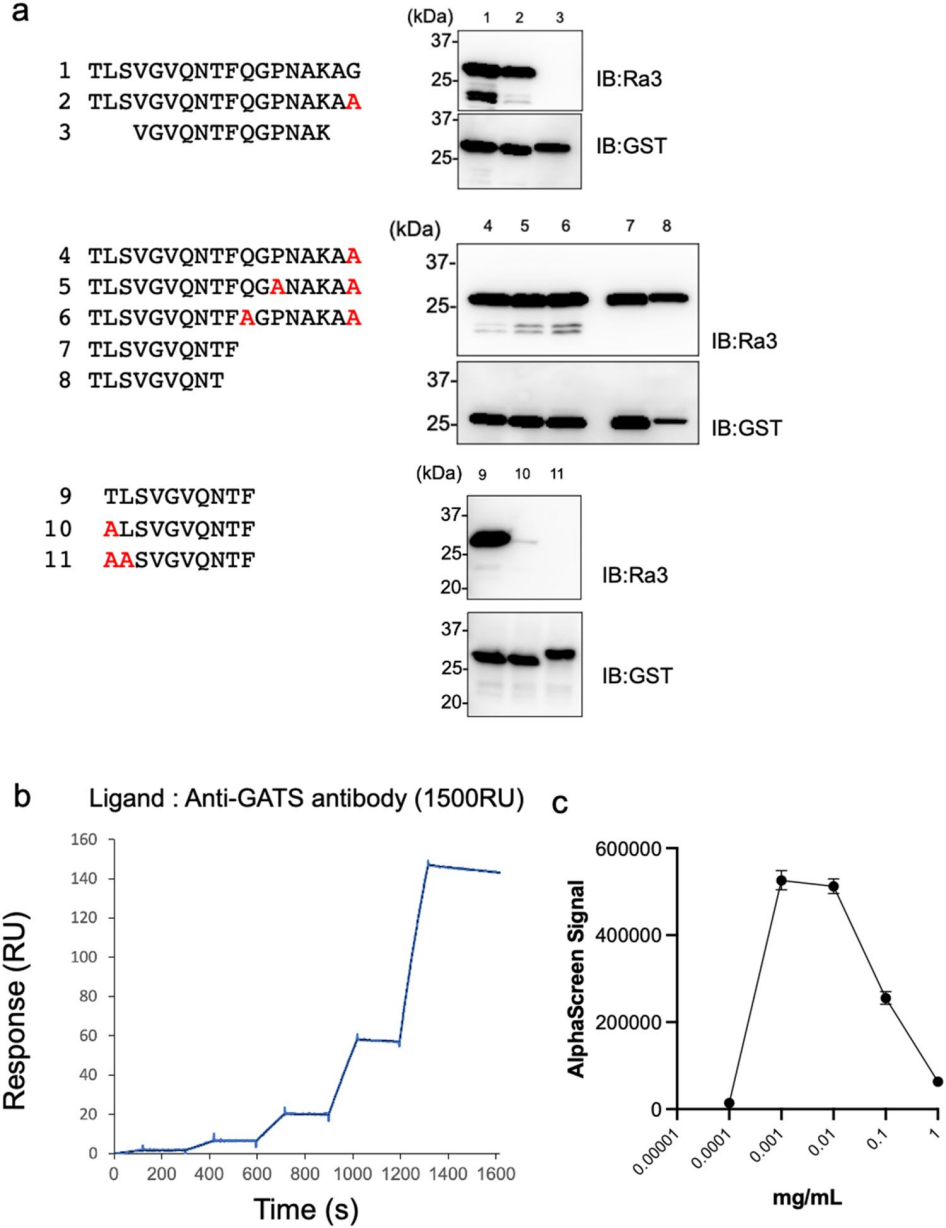
The minimal amino acid epitope determination of Ra3. (**a**) Immunoblot analysis of deletion mutants to identify the minimum Ra48 epitope sequence. (**b**) Kinetics assay of the GATS tag and anti-GATS antibody. Anti-GATS antibody was captured on a protein G-immobilized Biacore sensorchip at 1500 RU. Purified FLAG-GST-GATS protein was then injected for 1600 s as analyte. Black lines represent a global fit of a 1:1 interaction model to each kinetic data set. (**c**) Pointing out antibody concentrations in AlphaScreen. The GATS tag was fused to the N-terminus of GST-TEV-bls protein and synthesized using a wheat germ cell-free system.

To determine the detailed epitope of the Ra3 mAb, 11 different sequences of the same form (GST-TEV-bls-fragment) were synthesized in the wheat cell-free system and used for immunoblotting (Fig. 3a). First, the Gly terminus was replaced with Ala to determine whether C-terminal Gly was required for recognition. The substituted fragments were also recognized by Ra3 mAb, suggesting that C-terminal Gly was not required for Ra3 mAb recognition. Using similar approaches, the smallest recognition sequence, TLSVGVQNTF, was determined to be the epitope tag. Fortunately, no lysine residues were found in this sequence. This epitope sequence was named the GATS tag. Hereafter, the Ra3 mAb is referred to as the anti-GATS antibody. Anti-GATS antibody was transiently expressed in Expi293F cells and purified from the medium using protein G-sepharose. Both heavy and light chains were examined using CBB staining (Supplementary Fig. 1). The purified anti-GATS antibody was used for all assays.

### Affinity detection of the GATS tag system and an optimal condition using AlphaScreen

To investigate the affinity of the GATS tag system consisting of the GATS tag and anti-GATS antibody, a FLAG-GST-GATS recombinant protein was synthesized using the wheat cell-free system and purified using glutathione-conjugated magnetic Sepharose. Affinity was measured by SPR using the purified FLAG-GST-GATS protein and anti-GATS antibody (Fig. 3b). When 1500 RU of anti-GATS antibody was captured on a sensorchip, kinetic analysis from 1500 RU yielded a K_D_ value = 8.71 × 10^–9^ M (ka = 1.07 × 10^4^ 1/Ms, kd = 9.32 × 10^–5^ 1/s).

Next, the anti-GATS antibody was used in the AlphaScreen method. A low amount of anti-GATS antibody (0.001 mg/mL) was sufficient for detection by AlphaScreen (Fig. 3c). This high affinity of the GATS system could be expected to provide highly sensitive detection for immunoblotting and other applications, and various GATS-fusion proteins synthesized in the wheat cell-free system could be detected by immunoblotting with low background (Supplementary Fig. Supplementary Fig. 2a).

### Performance of the GATS Tag System in Analysis of Cell Biology

**Figure 4:**
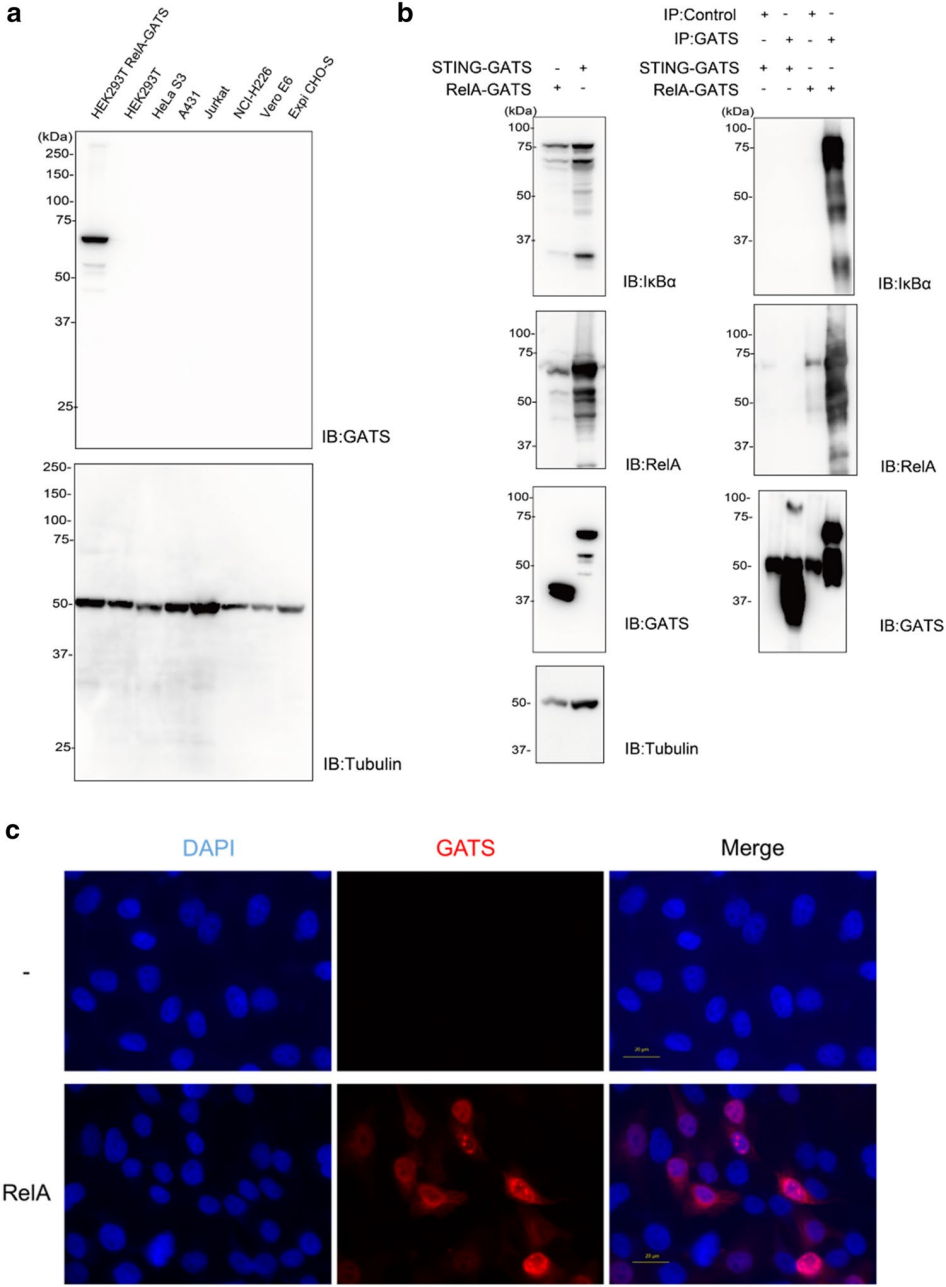
Use of anti-GATS antibodies for specificity assessment. (**a**) Specificity assessment of anti-GATS antibodies using various cell lysates. The dilution rate of GATS antibodies is 0.2 µg/mL. (**b**) Co-immunoprecipitation assay using the GATS tag system. (**c**) Immunostaining use of the RelA-targeted GATS tagging system.

In cell biology, many studies use peptide tag systems. The GATS tag system was used for cellular analysis. We used RelA to validate the ability of the GATS tag system because it is well characterized and is widely known to interact with IκBα^22,23^. A GATS tag was fused to the C-terminus of RelA (RelA-GATS) and was subsequently expressed in HEK293T cells. The cross-reactivity of the anti-GATS antibody was also validated against human cell lysates from HEK293T, HeLa S3, A431, Jurkat, and NCI-H226 cells, except for Vero E6 (African green monkey) and Expi CHO-S (Chinese hamster) cells. Immunoblotting showed that RelA-GATS was clearly detected, and no extra bands were found in normal cell lysates (IB: GATS in Fig. 4a), indicating that the GATS tag system provided highly sensitive detection in mammalian cell lysates with low background. The detection of FLAG-GST-Venus synthesized by the wheat cell-free system using the anti-GATS antibody showed a lower background with the anti-GATS antibodies than with FLAG and GFP antibodies (Supplementary Fig. 2b). HEK293T cells overexpressing STING-GATS and RelA-GATS were detected with GATS antibodies and HRP-fused GATS antibodies. The use of HRP-fused anti-GATS antibodies resulted in a lower background than that when GATS antibodies alone were used (Supplementary Fig. 2c).

The tag system has also been used extensively in immunoprecipitation assays. Thus, the GATS tag system was used in immunoprecipitation assays. Using the well-known interaction pair of RelA and IκBα^22,23^, the GATS tag system was validated in a co-immunoprecipitation assay. GATS-tagged RelA was expressed in HEK293T cells stably expressing IκBα-TurboID^4^, and cell lysates were immunoprecipitated with anti-GATS antibody and Protein A -Dynabeads. As a negative control, STING-GATS was expressed, and general IgG was used. Immunoblot analysis showed that endogenous IκBα and IκBα-TurboID co-immunoprecipitated with RelA-GATS (Fig. 4b). GATS-STING was also precipitated by the anti-GATS antibody but not by IκBα. These results indicated that the GATS tag system is specifically applicable to immunoprecipitation assays.

Determining the subcellular localization of a target protein by immunostaining is essential for understanding the biological function of the protein. To investigate whether the GATS tag system could be used for immunostaining, RelA-GATS was expressed in HeLa cells. After fixation and permeabilization, RelA-GATS was visualized using an anti-GATS antibody and fluorescence-labelled secondary antibody (Fig. 4c). Similar to previous reports^23^, RelA-GATS was mainly found in the nucleus with low background staining. Taken together, these results indicate that the GATS tag system is suitable for cell biology analysis.

### Development of the GT7 tag for protein purification based on the GATS system

**Figure 5:**
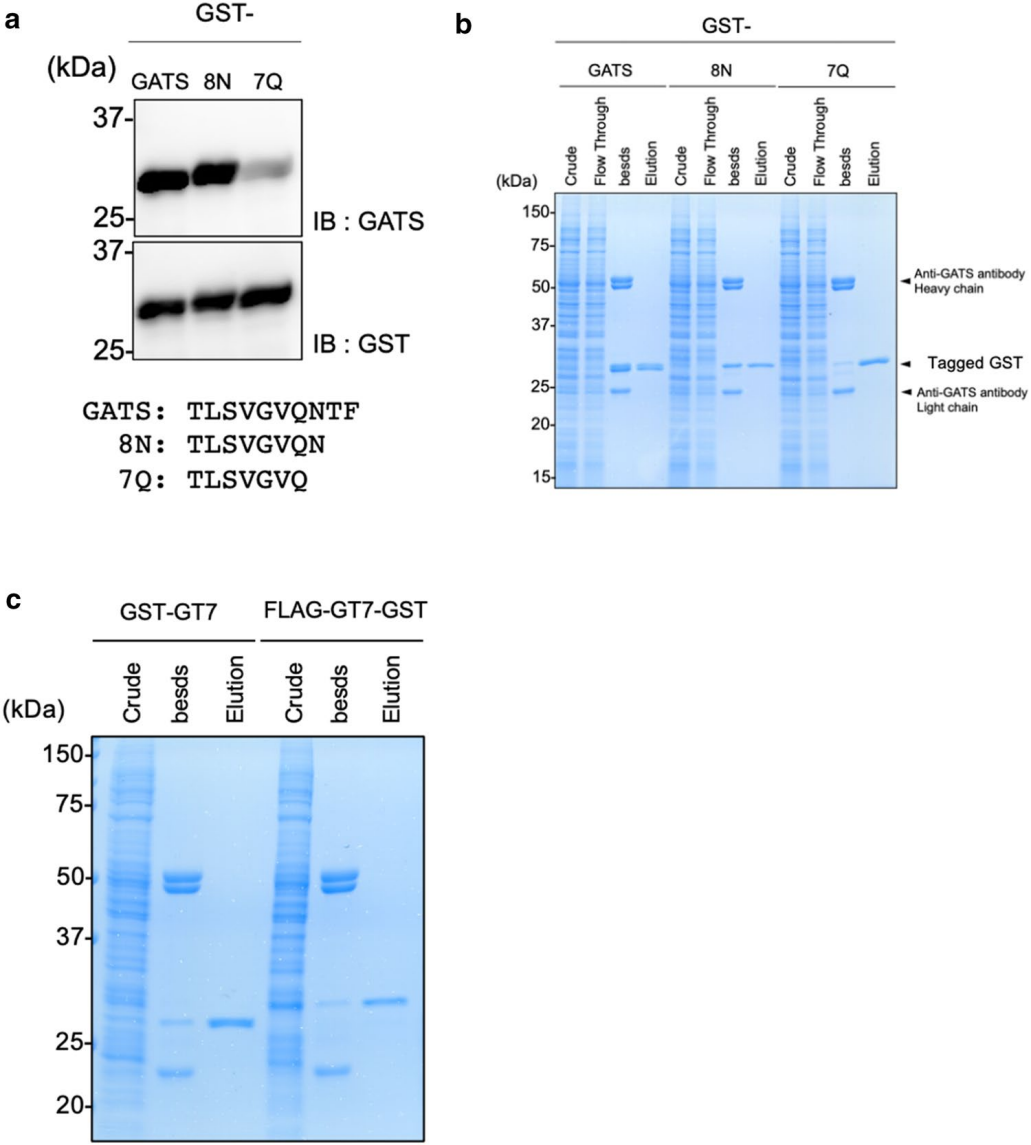
Development of the protein purification tag GT7. (**a**) Binding confirmation of GATS tag mutant to anti-GATS antibody by immunoblotting. Antigen proteins were synthesized by a wheat cell-free protein synthesis system. (**b**) Purification of GATS-tag or GATS-tag mutant fusion GST by GATS peptides. Eluted proteins and proteins on sepharose were confirmed by CBB staining. (**c**) Purification of N-terminally GT7-tagged GST protein by GATS peptides. Eluted proteins and proteins on sepharose were confirmed by CBB staining.

Purification of target proteins using antibodies is an important technique in biochemistry and structural biology. Therefore, we developed a protein purification method based on the GATS-tag system. A short sequence of the GATS sequence was deleted to improve the GATS system for protein purification. First, we generated 8N and 7Q mutants by trimming the C-terminus of the GATS tag into the GST-fusion form (Fig. 5a). The results indicated that the detection sensitivity of the anti-GATS antibody slightly decreased in 7Q mutants, that of 8N mutants was recognized to be almost the same level as the GATS tag. Second, C-terminally GATS tags, 8N- or 7Q-fused GST, were purified with protein G-conjugated beads to which anti-GATS antibodies were conjugated. Subsequently, the tag-fused GST proteins were eluted with GATS peptide (final concentration 150 µM). The quantity and quality of purified proteins were confirmed by CBB staining after SDS-PAGE. The results showed that the 7Q tag had the highest elution efficiency, whereas most of the GATS- or 8N tag fusion proteins remained on the beads (Fig. 5b). Because the 7Q tag could be used for protein peptide elution, we named it the GT7 tag from the GATS-based 7 amino acid tag. To further investigate whether the GT7 tag works at the N-terminus in protein purification, the GT7 tag was fused to the N-terminus of the GST protein (GT7-GST), and the GT7-GST was clearly eluted by the GATS peptide (Fig. 5c). Taken together, these results indicate that the GT7 tag system can be used for protein purification in the form of both N- and C-terminal fusions.

### GATS tagging system for the detection of biotin labelling proteins by NHS-biotin, BioID enzymes and HRP

**Figure 6:**
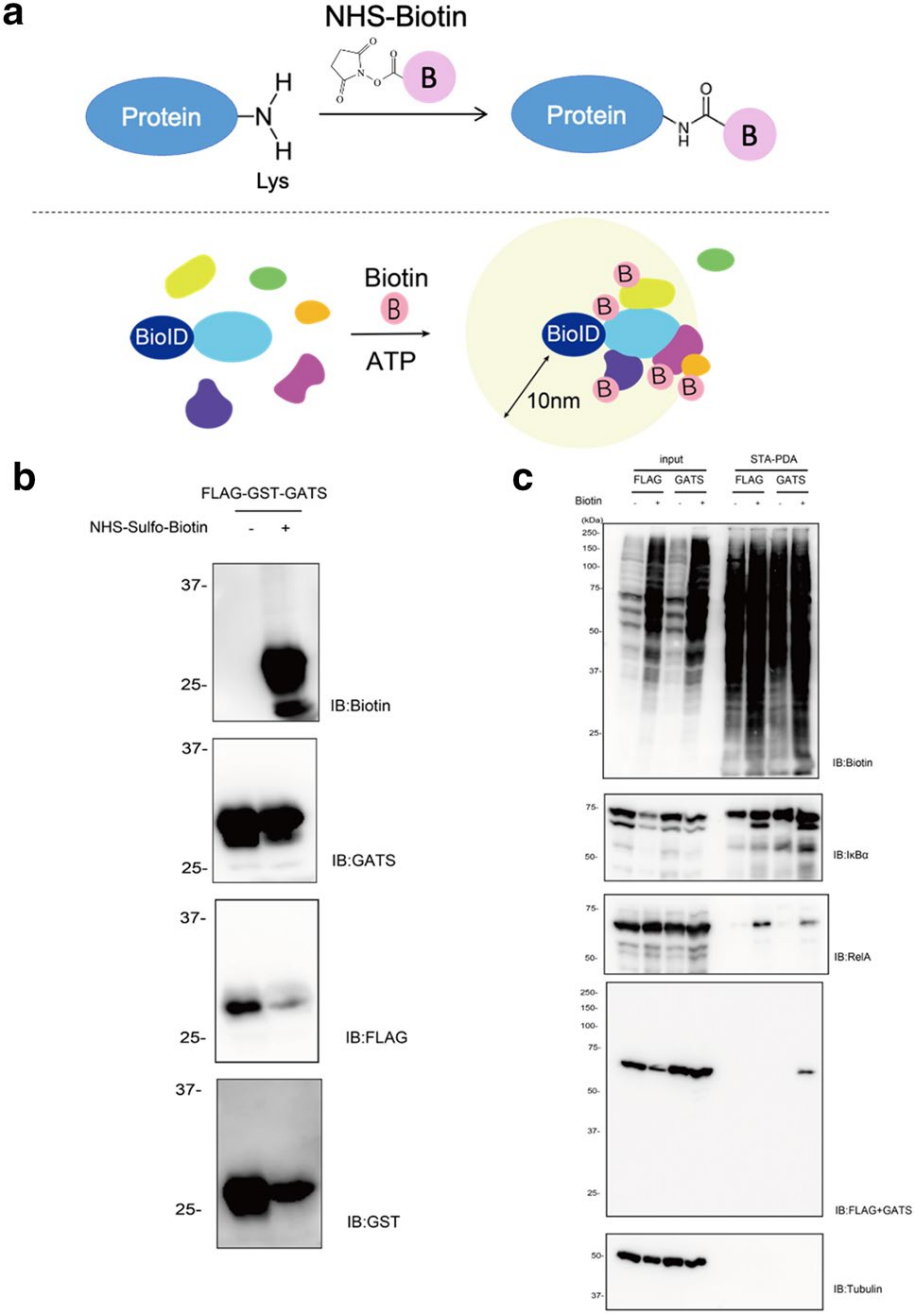
Application of GATS tags to biotin labellings by NHS-biotin and BioID methods. (**a**) Schematic diagram of the proximity labelling method using NHS-biotin (upper panel) and the BioID method (lower panel). (**b**) Biotin labelling of GATS-tag fusion proteins using NHS-Sulfo-Biotin and functional check of the GATS tag. Purified FLAG-GST-GATS treated with NHS-Sulfo-Biotin. The recognition of antibodies was evaluated using immunoblotting. (**c**) Streptavidin pull-down assay. pCAGGS-FLAG-RelA or pcDNA3.1-AGIA-RelA-GATS were transfected in HEK293T cells stably expressing AGIA-TurboID-IκBα. After transfection for 24 h, D-biotin was added to a 50 µM culture medium for 2 h. The biotinylated proteins were then pulled down using streptavidin beads and analyzed by immunoblotting.

NHS-biotin has been widely used for biotin labelling of proteins^24,25^. Because the NHS-ester reacts with amino groups of proteins such as lysine residues and the N-terminal (upper panel in Fig. 6a), a tag system without lysine residues would be suitable for protein detection after the NHS -ester reaction. Fortunately, the epitope of the GATS tag is TLSVGVQNTF, which has no lysine residue. To investigate the detection ability of the GATS tag system after the sulfo-NHS-biotin reaction, a double-tagged protein of FLAG-GST-GATS was used, where FLAG or GATS tags were fused to the N- or C-terminus of the GST protein, respectively. The GATS tag system adequately detected FLAG-GST-GATS after biotin labelling with NHS-biotin (Fig. 6b), whereas anti-FLAG and anti-GST antibodies reduced the detection sensitivity by less than half.

BioID technology is widely used to identify partner proteins^1,2^. BioID generally has promiscuous activity and releases highly reactive and short-lived biotinoyl-5′-AMP. The released biotinoyl-5′-AMP modifies lysine residues on proximal proteins (within a distance of 10 nm) (lower panel in Fig. 6a)^2^. Similar to the detection of biotin labelling by NHS-biotin, the presence of lysine residues within the tag would likely result in biotin labelling of the tag and thus, reduce the reactivity of the antibody. The GATS tag system was thus used for BioID. RelA-FLAG and RelA-GATS were also overexpressed and biotinylated in HEK293T cells stably expressing TurboID-IκBα. The biotinylated RelA and IκBα proteins were recovered by streptavidin-pull down assay (STA-PDA) and detected by FLAG or GATS antibody. Immunoblotting showed that GATS could detect the RelA protein after STA-PDA (arrowhead in Fig. 6c), whereas no band was detected with the FLAG antibody. Taken together, these results indicate that the GATS tag system is suitable for the detection of biotin labelling methods such as BioID.

APEX2 and HRP activate tyramides in the presence of H_2_O_2_ to produce short-lived radicals that can react with electron-rich amino acids such as tyrosine^26^. This reaction has been widely used to identify protein–protein interaction by proximity biotinylation labeling using the biotinyl tyramide^27,28^. However, the biotinylation reaction using APEX2 and HRP results in the biotinylation of tyrosine, and the use of a peptide tag with tyrosine would reduce the reactivity of the antibody. Therefore, we verified whether GATS tags without a tyrosine tag are useful for proximal biotinylation labeling using peroxidase. RelA-HA and RelA-GATS were overexpressed and lysed in HEK293T cells, the lysate was then biotinylated with HRP (Supplementary Fig. 3). After biotinylation, the reactivity of the tags was tested by immunoblotting. Immunoblotting results showed that the reactivity of the HA tag with anti-HA antibodies weakened after biotinylation with HRP (Supplementary Fig. 3). On the other hand, GATS antibodies recognized RelA-GATS, and a part of the RelA-GATS after biotinylation reaction was shifted to the high molecular weight side. This indicates that the GATS antibody recognizes RelA-GATS whose electrophoretic mobility was altered by biotinylation. These results suggest that the GATS tag, which does not contain tyrosine residues in the tag sequence, is also useful for proximity biotinylation labeling using peroxidase.

## Discussion

In this study, we developed a highly specific GATS-tag system. Polypeptide tag systems have become important tools for the analysis of protein functions in biochemistry, cell biology, and molecular biology. For example, polypeptide tag systems significantly contribute to immunoblotting, immunostaining, AlphaScreen, and immunoprecipitation assays. The immunoblotting assay with the GATS tag system was highly sensitive in the wheat cell-free and mammalian cultured cell systems used (Fig. 4 and Supplementary Fig. 2). Immunostaining revealed a low background signal due to non-specific binding (Fig. 4c). Furthermore, the AlphaScreen method allows highly sensitive detection at a final concentration of 0.025 ng per assay (25 µL) (Fig. 3c). In immunoprecipitation, the GATS tag successfully co-precipitated complex proteins without affecting the protein interactions (Fig. 4b). Thus, the GATS tag is a highly sensitive tag antibody with low non-specific binding.

The anti-GATS antibody has a very strong binding affinity to the GATS tag with a Kd of 8.71 × 10^–9^ M (Fig. 3b). The GATS tag system is a rabbit monoclonal antibody-based tag system using the ISAAC method^29^. ISSAC method has been used in our previous work to develop AGIA tags and CP5 tag systems that recognize epitopes of the human dopamine receptor DRD1. AGIA and CP5 have been developed using the rabbit monoclonal antibody system^30,31^. AGIA and CP5 are tag antibodies with high specificity, and the high specificity of the GATS tag in this case also indicates the usefulness of rabbit monoclonal antibodies.

The GATS tag system was designed using an approach that ensures minimal nonspecific binding, a problem with existing tag systems. The GATS tag system uses an antibody against PfGAMA, a unique proteinof *P. falciparum* malaria parasite, derived from malarial micronemes. Micronemes are intracellular organelles found in the cell-invasive *Plasmodium falciparum* of the phylum Apicomplexa and are therefore not present in mammalians and plants^32^. This approach has succeeded in minimizing non-specific binding to proteins in mammalian cells (Fig. 4a). This approach of using proteins localized to cellular organelles specific to a particular organism as antigens provides valuable information for future tag development.

Another unique feature of the GATS tag system is that tag sequences do not contain lysine residues, which makes it suitable for use in biotin labelling methods such as BioID. Many protein interactions are currently being analyzed using the BioID method^5^. The BioID method allows biotin labelling of lysine residues of proteins in close proximity by fusing BioID enzymes to target proteins, enabling a comprehensive analysis of interacting proteins^1,2^. A pull-down method using streptavidin beads was used to confirm the biotinylation of the target and interacting proteins. The use of tags containing lysine in this process can make detection with tagged antibodies difficult. Moreover, in this study, it was difficult to detect biotinylated proteins with the FLAG tag, while the GATS tag without a lysine residue could detect them (Fig. 6c). AGIA and HA tags do not contain lysine residues, but multiple tag antibodies are very useful when observing biotinylation of the protein complex. Therefore, the development of the GATS tag system is expected to contribute to the development of the BioID method. NHS-based protein labelling has been widely used for labelling purified proteins^24,25^. The GATS tag system would also be suitable for protein detection after NHS ester labelling. We have previously reported the use of TurboID to develop a simple protein biotinylation method^33^. Thus, the need for biotinylated labelling of proteins is increasing, and the choice of tags is important. In summary, the GATS tag system provides a novel tag system for biotin labelling methods and protein purification.

## Materials and methods

### Antibodies

The following HRP-conjugated antibodies were used in this study: FLAG (Sigma-Aldrich, A8592,1:5000), AGIA (produced in our laboratory), α-tubulin (MBL, PM054-7, 1:5000), GST (M071-7, 1:5000), and biotin (Cell Signaling Technology, #7075, 1:1000). The following primary antibodies were used in this study: IκBα (CST L35A5, 1:1000), RelA (CST L8F6, 1:1000), GFP (MBL, M048-3 1:1000), HSP90 (CST, #4874, 1:1000), Ra mAb (produced in our laboratory), and GATS (produced in our laboratory, 0.2 µg/mL). Anti-rabbit IgG (HRP-conjugated, Cell Signaling Technology, #7074, 1:10,000) and anti-mouse IgG (HRP-conjugated, Cell Signaling Technology, #7076, 1:10,000) were used as secondary antibodies.

### Plasmid constructions

All the primer sequences used in this study are listed in Supplementary Table 1. The amino acid sequences of the GAMA and Ripr used are described in Supplementary Table 2. pcDNA3.1, or pCAGGS vectors were purchased from Invitrogen or RIKEN, respectively. The pEU-based expression vectors, including pEU-E01-FLAG-GST-TEV-MCS, pEU-E01-MCS, pEU-E01-MCS-bls, pEU-E01-FLAG-GST-MCS, pEU-E01-FLAG-MCS and pEU-E01-GST-TEV-bls-MCS were used for wheat germ cell-free protein synthesis. pcDNA3.1-AGIA-MCS and pCAGGS-FLAG-MCS vectors are used for mammalian cell expression. For the GAMA and Ripr genes, we used those cloned in previous studies^19,21^. The ORFs STING, RelA, CRBN were purchased from the Mammalian Gene Collection (MGC). The restriction enzyme sites were added to GAMA-F, Ripr-F, GAMA-F fragments, GATS sequence, GT7 sequence and RelA by PCR. GAMA-F and Ripr-F were cloned into pEU-E01-MCS-bls. GAMA-F fragments, GATS sequence and GT7 sequence were cloned into pEU-E01-GST-TEV-bls-MCS. GATS sequence was cloned into pEU-E01-FLAG-GST-MCS. RelA was cloned into pCAGGS-FLAG-MCS. The restriction enzyme sites and GATS sequence were added to STING, RelA, CRBN and Venus by PCR. CRBN and Venus were cloned into pEU-E01-MCS. Venus was cloned into pEU-E01-FLAG-MCS. STING, RelA were cloned into pcDNA3.1-AGIA-MCS. The GT7 sequence was cloned into pEU-E01-FLAG-GST-TEV-MCS using Gibson Assembly (New England Biolabs).

### Cell culture and transfection

HEK293T and HEK293T cells stably expressing AGIA-AirID-IκBα^4^ and HeLa cells were cultured in low-glucose DMEM (FUJIFILM Wako Pure Chemical Corporation) supplemented with 10% fetal bovine serum (FUJIFILM Wako), 100 U/mL penicillin, and 100 µg/mL streptomycin (Gibco, Thermo Fisher Scientific) at 37 °C under 5% CO_2_. HEK293T cells, HEK293T cells stably expressing AGIA-AirID-IκBα, and HeLa cells were transiently transfected with polyethyleneimine (PEI) Max (MW 40,000) (PolyScience, Inc.).

Expi293F cells (Gibco, Thermo Fisher Scientific) were centrifuged at 37 °C and 8% CO_2_ at 125 ± 5 rpm in Expi293F medium (Gibco, Thermo Fisher Scientific) supplemented Antibiotic–Antimycotic (50 U/mL of penicillin, 50 µg/mL of streptomycin, and 0.5 µg/mL of Gibco Amphotericin B) (Gibco, Thermo Fisher Scientific).

### Antibody production and purification

cDNAs for the Ra clone antibody heavy and light chains were subcloned into the pcDNA3.4 expression vector using PCR and In-Fusion Reaction. The Ra clone antibody was expressed using the Expi293F Expression System (Gibco, Thermo Fisher Scientific), according to the manufacturer’s instructions. The antibody secreted in the culture medium was purified using protein G Sepharose 4 Fast Flow (GE Healthcare), and then buffer exchange was dialyzed in phosphate buffered saline (PBS). The purified antibody was frozen and stored at − 80 °C.

### Protein synthesis by a wheat cell-free protein production system

In vitro transcription and wheat cell-free protein synthesis were performed using the WEPRO1240 expression kit (Cell-Free Sciences). A transcript was generated from each of the DNA templates using SP6 RNA polymerase. The translation reaction was performed using a WEPRO1240 expression kit (Cell-Free Sciences). For biotin labelling, 1 μL of BirA or ancestral BirAs produced by the wheat cell-free expression system was added to the bottom layer, and 500 nM (final concentration) of D-biotin (Nacalai Tesque) was added to both the upper and bottom layers, as described previously^34^. The GAMA-F used for the rabbit immunization was expressed by WEPRO7240 expression kit (Cell-Free Sciences) and purified with strep-Tactin® Sepharose® (IBA, Göttingen, Germany) as per manufacturer’s instructions.

### Immunoblotting

Immunoblotting was performed according to the standard protocols. Briefly, proteins in whole-cell lysates were subjected to sodium dodecyl sulfate–polyacrylamide gel electrophoresis (SDS-PAGE) and transferred onto a polyvinylidene fluoride (PVDF) membrane by wet blotting. After blocking with 5% milk/TBST or 5% BSA/TBST, the membranes were tested using the indicated antibodies and horseradish peroxidase (HRP)-conjugated antibody. All immunoblot data were analysed using ImageJ software. The original blots are presented in Supplementary Figs. 4-9.

### AlphaScreen-based biochemical assays using recombinant proteins

All recombinant proteins were synthesized using a wheat germ cell-free synthesis system. Antibody binding was detected using an AlphaScreen IgG (Protein A) detection kit (Perkin Elmer). Briefly, 25 μL of detection mixture containing 1 µL of GST-TEV-bls fused recombinant proteins, 100 mM Tris–HCl (pH 8.0), 0.1% Tween 20, 100 mM NaCl, 10 ng of anti-GATS antibody (produced in our laboratory), 1 mg/mL BSA, 0.08 μL of streptavidin-coated donor beads, and 0.08 μL of protein A-conjugated acceptor beads was added to each well of an Optiplate 384 titer plate (PerkinElmer) before incubation at 26 °C for 1 h. Luminescence signals were detected using the AlphaScreen detection program with an EnVision device (PerkinElmer). All AlphaScreen signal values are listed in Source data file.

### Protein purification of GST and GT7-tagged protein

Crude FLAG-GST-GATS recombinant protein (1.5 mL) was synthesized using a wheat germ cell-free reaction. The protein synthetic solution was mixed with 30 µL of MagneGST™ Glutathione Particles (Promega) previously equilibrated with PBS and rotated at 4 °C for 1 h. After the flow-through solvent was removed, the beads were washed thrice with 1 mL PBS. Recombinant proteins were eluted with 55 µL of elution buffer (10 mM reduced GSH, 50 mM Tris–HCl pH 8.0, and 50 mM NaCl).

Purification of GT7-tagged proteins using high-concentration GATS peptides was performed as follows. 10 µg of GATS antibody and 10 µL of protein A Sepharose were added and mixed at 4 °C for 1 h with rotation. Sepharose was transferred to Pierce™ Micro-Spin Columns (Thermo Fisher Scientific) and washed thrice with 1 mL of PBST. Next, 20 µL of PBS containing 150 µM GATS peptide (EEAAGIARPLIATLSVGVQNTF) (GenScript) was added to the column. After incubation at room temperature for 1 h, the eluted fractions were collected via centrifugation. Finally, Sepharose was mixed with 20 μL of SDS-PAGE sample buffer and incubated at 99 °C for 5 min to analyze proteins that were not eluted by peptide treatment. All fractions were confirmed by SDS-PAGE and CBB staining. The original blots are presented in Supplementary Fig. 6.

### Biacore assay

Biacore experiments were conducted using the Biacore X100 apparatus (GE Healthcare). HBS-EP + (10 mM HEPES–NaOH (pH 7.4), 150 mM NaCl, 0.05% Tween 20, and 3 mM EDTA) was used as running buffer. The temperature of the flow cells was kept at 25 °C.

A kinetic assay was performed using the capture method. anti-GATS antibody was immobilized on a CM5 sensor chip by amine coupling at 1500 RU to capture the antibodies. FLAG-GST-GATS protein was synthesized using a cell-free system and purified using glutathione sepharose. The concentration of the GST-purified FLAG-GST-GATS and Protein G-purified antibody was assayed by the extinction coefficient method using a NanoDrop spectrophotometer (Thermo Fisher Scientific). The extinction coefficient was calculated using ProtParam (http://web.expasy.org/protparam/)^35^. All single-cycle was performed according to manual mode. Flow rate was 30 μl/min, contact time was 120 s, and dissociation time was 300 s. The affinity parameter was calculated by using BiaEvaluation software (Cytiva).

### Immunostaining

Transfected HeLa cells were fixed with 4% paraformaldehyde in phosphate-buffered saline (PBS) for 5 min at room temperature and then permeabilized with 0.5% Triton X-100 in PBS for 5 min. After blocking with 5% calf serum in TBST for 1 h, cells were incubated with anti-GATS antibody overnight at 4 °C. After washing with TBST, the cells were incubated with F(ab')2-Goat anti-Rabbit IgG (H + L) Cross-Adsorbed Secondary Antibody, Alexa Fluor™ 555 (Invitrogen, Thermo Fisher Scientific) for 1 h at room temperature. Nuclear staining was counterstained with 4,6-diamidino-2-phenylindole (DAPI). After a final wash with TBST, the coverslips were mounted with an antifade. Images were taken using an all-in-one fluorescence microscope BZ-X810 (KEYENCE).

### Immunoprecipitation

Each gene was transfected into cells stably expressing AGIA-TurboID-IκBα, which were cultured in a 10-cm dish. After incubation for 24 h, cells were harvested using a cell scraper. The cell pellets were washed with 1 mL of 1 × PBS buffer and lysed with 1 mL of IP lysis buffer (25 mM Tris–HCl pH 7.5, 150 mM NaCl, 1 mM EDTA, 1% NP-40, and 5% glycerol) with protease inhibitors (Roche), and the lysates were rotated at 4 °C for 30 min. Then, 970 µL of the lysate was added to 50 µL of IP lysis buffer containing 10 µL of protein A Dynabeads (Thermo Fisher Scientific), rotated at 4 °C for 30 min, and the supernatant was collected. For immunoprecipitation, the supernatant was added to 1 µg of the anti-GATS antibodies and rotated at 4 °C for 2 h. Then, the supernatant was added to 50 µL of IP lysis buffer containing 10 µL of protein A Dynabeads (Thermo Fisher Scientific) and rotated at 4 °C overnight. After washing twice with 1 mL of 1 × PBS and once with 1 mL of IP lysis buffer, the immunocomplexes were boiled in 40 µL SDS sample buffer containing 5% 2-mercaptoethanol. The boiled solution was analyzed using SDS-PAGE and immunoblotting. The original blots are presented in Supplementary Fig. 5.

### In vitro chemical biotin labelling

GST-purified FLAG-GST-GATS (10 µL) was added to 10 mM EZ-Link Sulfo-NHS-Biotin (Thermo Fisher Scientific) in PBS (2.2 µL) and incubated at 37 °C for 30 min. The reaction was quenched by the addition of 1 M glycine (final 1.22 µM) and incubated at room temperature for 30 min. The mixture was mixed with an equal volume of 2 × SDS sample buffer containing 5% 2-mercaptoethanol. The boiled solution was analyzed using SDS-PAGE and immunoblotting. The original blots are presented in Supplementary Fig. 7.

### Streptavidin pull-down assay (STA-PDA)

To prepare STA-PDA, HEK293T cells stably expressing AGIA-TurboID-IκBα were cultured in a 10-cm dish. Each gene was transfected into the cells. After incubation for 24 h, cells were harvested by suspension in TrypLE Select (Gibco). The pellets were resuspended in low-glucose DMEM without FBS, and D-biotin (final concentration 50 µM) was added and incubated at 37 °C for 6 h. After centrifugation, the supernatant was removed, and the cell pellets were washed with 1 mL of 1 × PBS buffer and lysed with 1 mL of lysis buffer [50 mM Tris–HCl (pH 7.5), 150 mM NaCl, and 1% sodium dodecyl sulfate (SDS)] containing a protease inhibitor cocktail; and the lysates were denatured by boiling at 98 °C for 15 min and sonication. Lysates were clarified by centrifugation at 16,100 × g for 15 min. The cell lysates (970 µL) were added to 200 µL of lysis buffer containing 20 µL of streptavidin Sepharose beads (GE Healthcare) and rotated at 27 °C for 1 h. The flow-through solvent was removed, the beads were washed three times using 1 mL of wash buffer (50 mM Tris–HCl [pH 7.5], 1% SDS, and 150 mM NaCl), and the beads were boiled in 40 µL of SDS sample buffer containing 5% 2-mercaptoethanol. The boiled solution was analyzed using SDS-PAGE and immunoblotting. The original blots are presented in Supplementary Fig. 7.

### Biotinylation assay by HRP

pCAGGS-RelA-HA or pcDNA3.1-AGIA-RelA-GATS were transfected into HEK293T cells which were cultured in a 24-well plate. After incubation for 24 h, cells were lysed in 50 µL of Lysis buffer (150 mM NaCl, 25 mM Tris–HCl [pH7.5], 1 mM EDTA, 1% Triton-X 100) with protease inhibitors (Roche). The cell lysate (35 µL) was added to 15 µL of HRP reaction buffer [33.3 µg/mL HRP (FUJIFILM Wako), 0.025% H_2_O_2_(FUJIFILM Wako), 33.3 µM Biotinyl tyramide(Merck)]. The reaction solution was incubated at 4 °C for 30 min. The mixture was mixed with an equal volume of 2 × SDS sample buffer containing 5% 2-mercaptoethanol. The boiled solution was analyzed using SDS-PAGE and immunoblotting.

The original blots are presented in Supplementary Fig. 9.

### Statistical analysis

The data are presented as mean ± standard deviation (SD) from more than three technical replicates. All experiment were done at least three times, Statistical analyses were performed with Prism9.

## Supplementary Information

Supplementary Figures.

Supplementary Table 1.

Supplementary Table 2.

## Data Availability

Methods were performed in accordance to the relevant guidelines and regulation. All data generated or analysed during this study are included in this published article, its supplementary information and Source data files. The information and data in this article are available from the corresponding author on reasonable request.
