# Supplementary material for: GATS tag system is compatible with biotin labelling methods for protein analysis
Source: Sci Rep. 2023 Jun 23;13:10243. doi: 10.1038/s41598-023-36858-y (PMC10290147; doi:10.1038/s41598-023-36858-y)
Supplement: Supplementary file 1 — Supplementary Figures. [file 41598_2023_36858_MOESM1_ESM.pdf]

GATS Tag System is compatible with Biotin Labelling Methods for Protein Analysis

Kohdai Yamada[1], Fumiya Soga[1], Soh Tokunaga[1], Hikaru Nagaoka[2], Tatsuhiko Ozawa[3], Eizo Takashima[2], Tatsuya Sawasaki[1*]

[1] *Division of Cell-Free Life Science, Proteo-Science Center, 3 Bunkyo-cho, Matsuyama, Ehime 790-8577, Japan.*

[2] *Division of Malaria Research, Proteo-Science Center, 3 Bunkyo-cho, Matsuyama, Ehime 790-8577, Japan.*

[3] *Department of Immunology, Faculty of Medicine, Academic Assembly, Advanced Antibody Drug Development Center, University of Toyama, Toyama 930-0194, Japan.*

*Corresponding Author:

Tatsuya Sawasaki

Proteo-Science Center, Ehime University, Matsuyama 790-8577, Japan

Tel: 81-89-927-8530

Fax: 81-89-927-9941

E-mail: sawasaki@ehime-u.ac.jp.

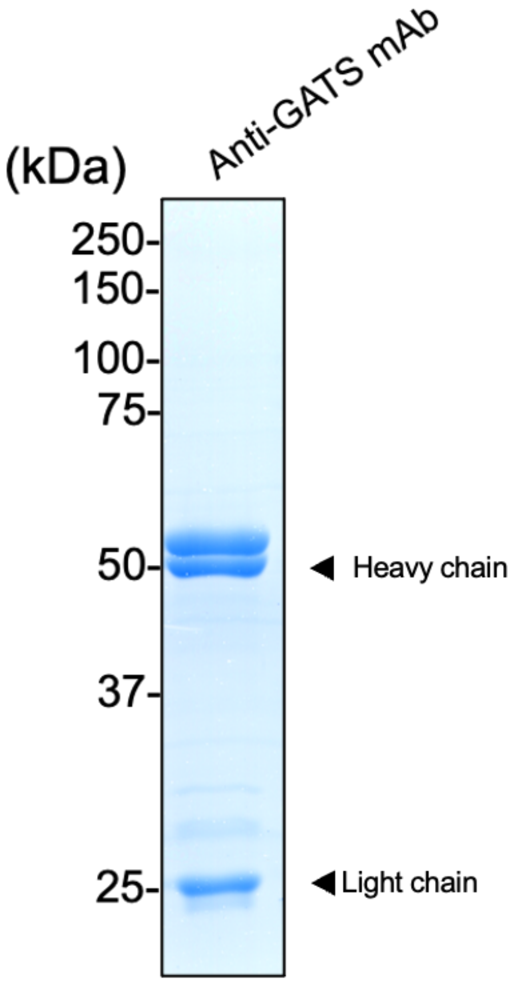

Supplementary Fig. 1 Purification of the anti-GATS antibodies

Transfection of Expi293F cells with plasmids coding GATS antibody and purification of GATS antibody using ProteinA Sepharose from cultures grown for one week.

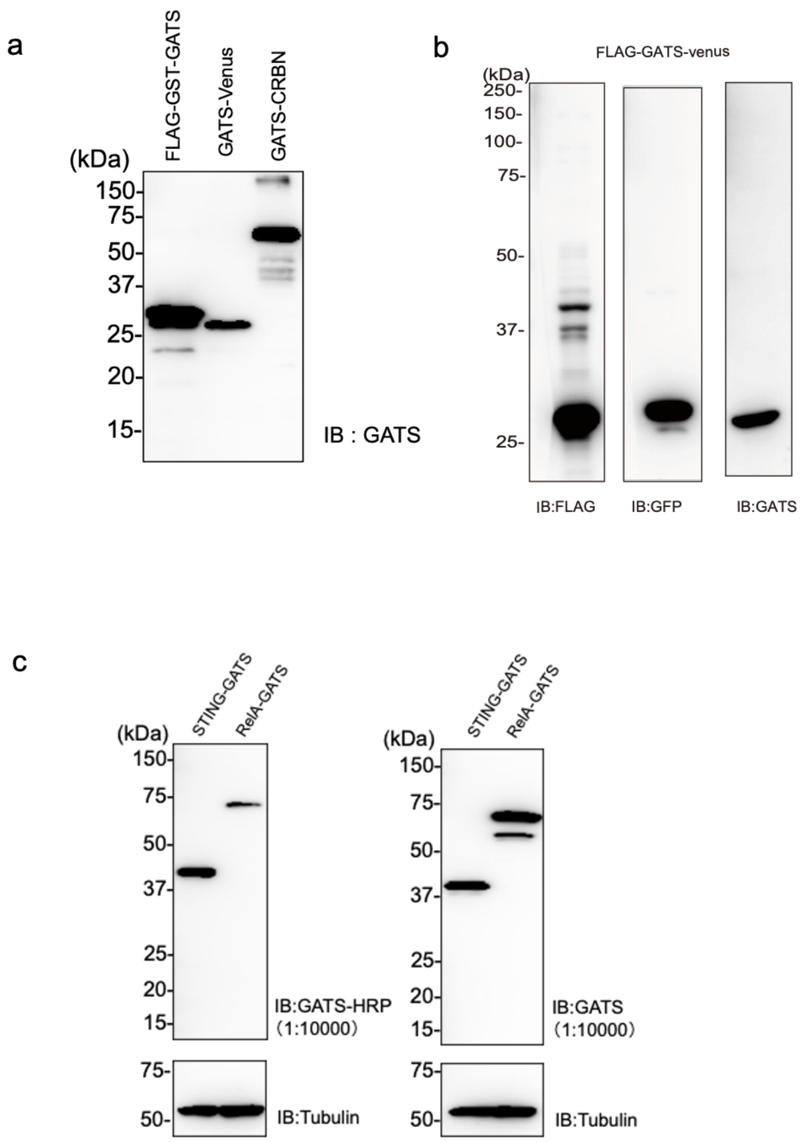

Supplementary Fig. 2 Immunoblotting detections using anti-GATS and HRP-conjugated anti-GATS antibodies

(a) Immunoblotting detection with anti-GATS antibodies of each protein synthesized using the wheat cell-free protein synthesis system. (b) Immunoblotting detection of FLAG-GATS-Venus by anti-FLAG and GFP, GATS antibodies. (c) Immunoblotting detection of STING-GATS- or RelA-GATS-expressing HEK293T cell lysates by anti-GATS- or HRP-fused anti-GATS antibodies. HRP-conjugated antibody was prepared using the HRP Conjugation Kit (Abcam).

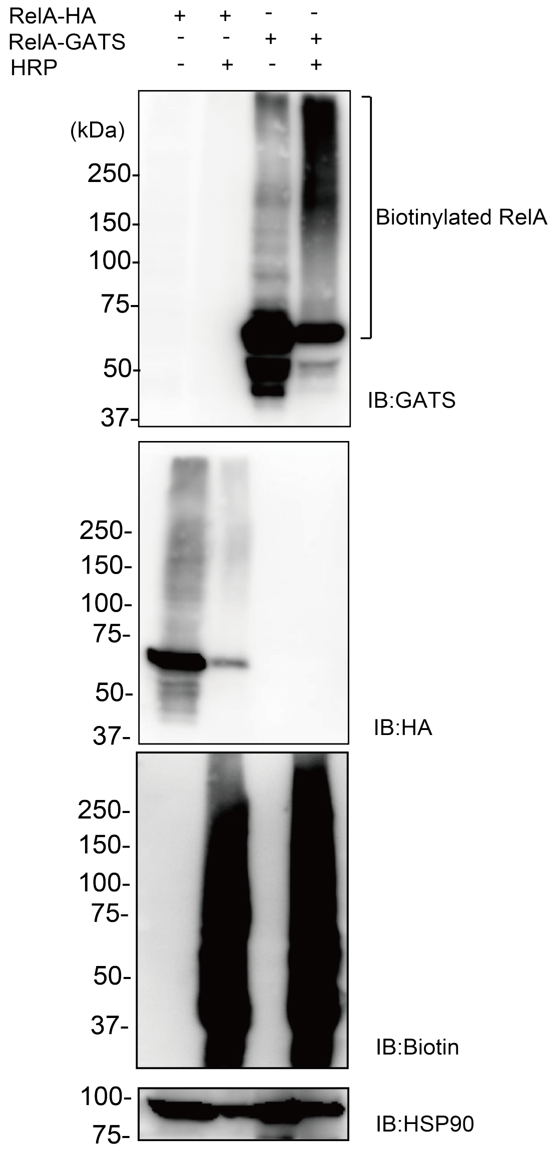

Supplementary Fig. 3 Detection of biotin labellings by the peroxidase using GATS tags.

The biotinylation assay by HRP. pCAGGS-RelA-HA or pcDNA3.1-AGIA-RelA-GATS were transfected into HEK293T cells which were cultured in a 24-well plate. After incubation for 24 h, biotinylation with HRP was performed using cell lysates and analyzed by immunoblotting.

Figure 1c

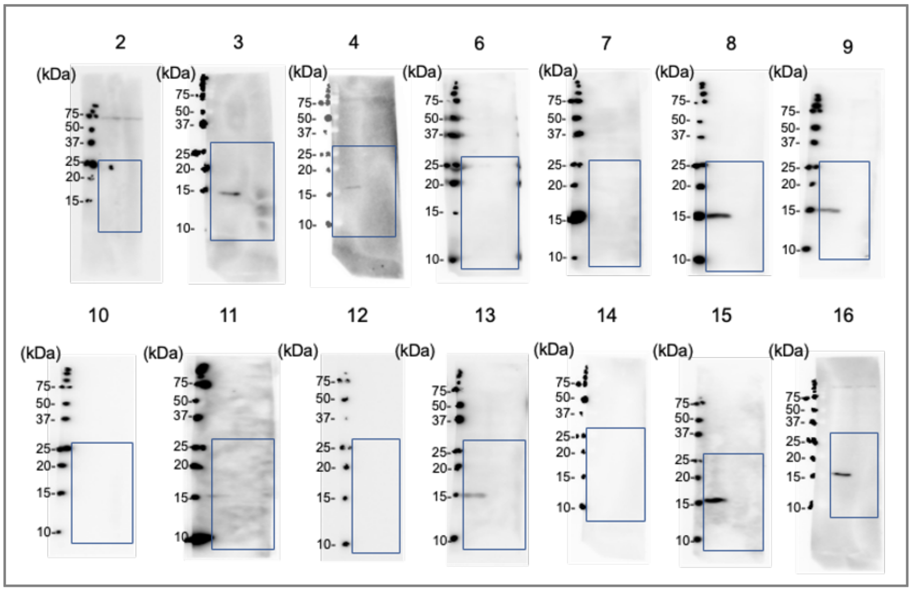

Figure 1e

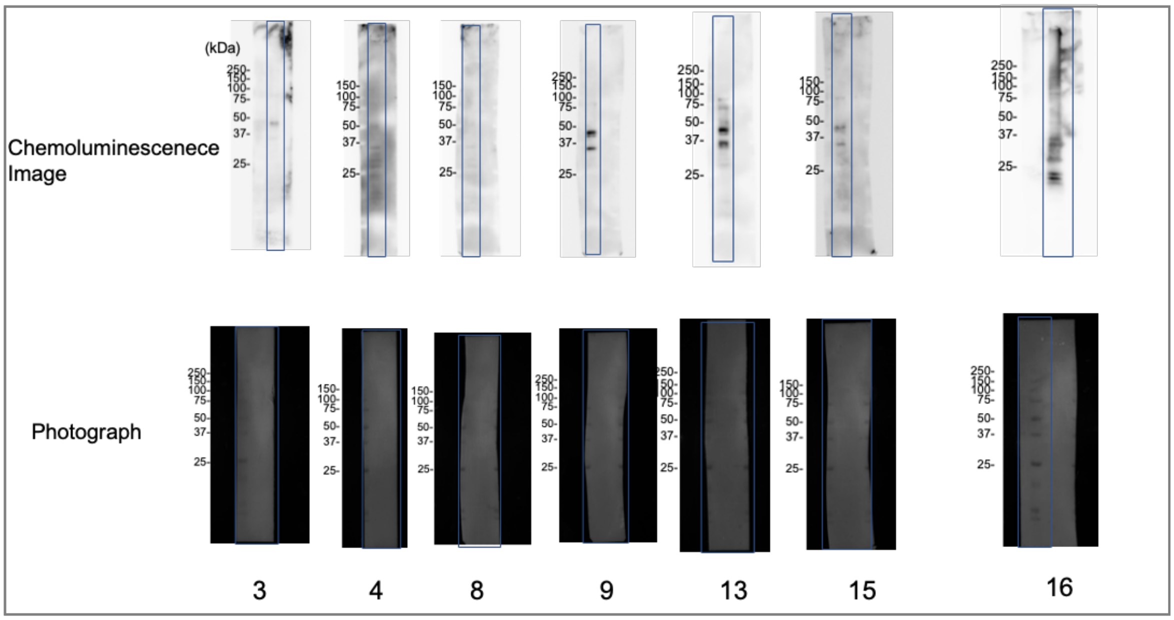

Supplementary Fig. 4 Full blot images of immunoblotting (Fig. 1)

All blots were performed using ImageJ.

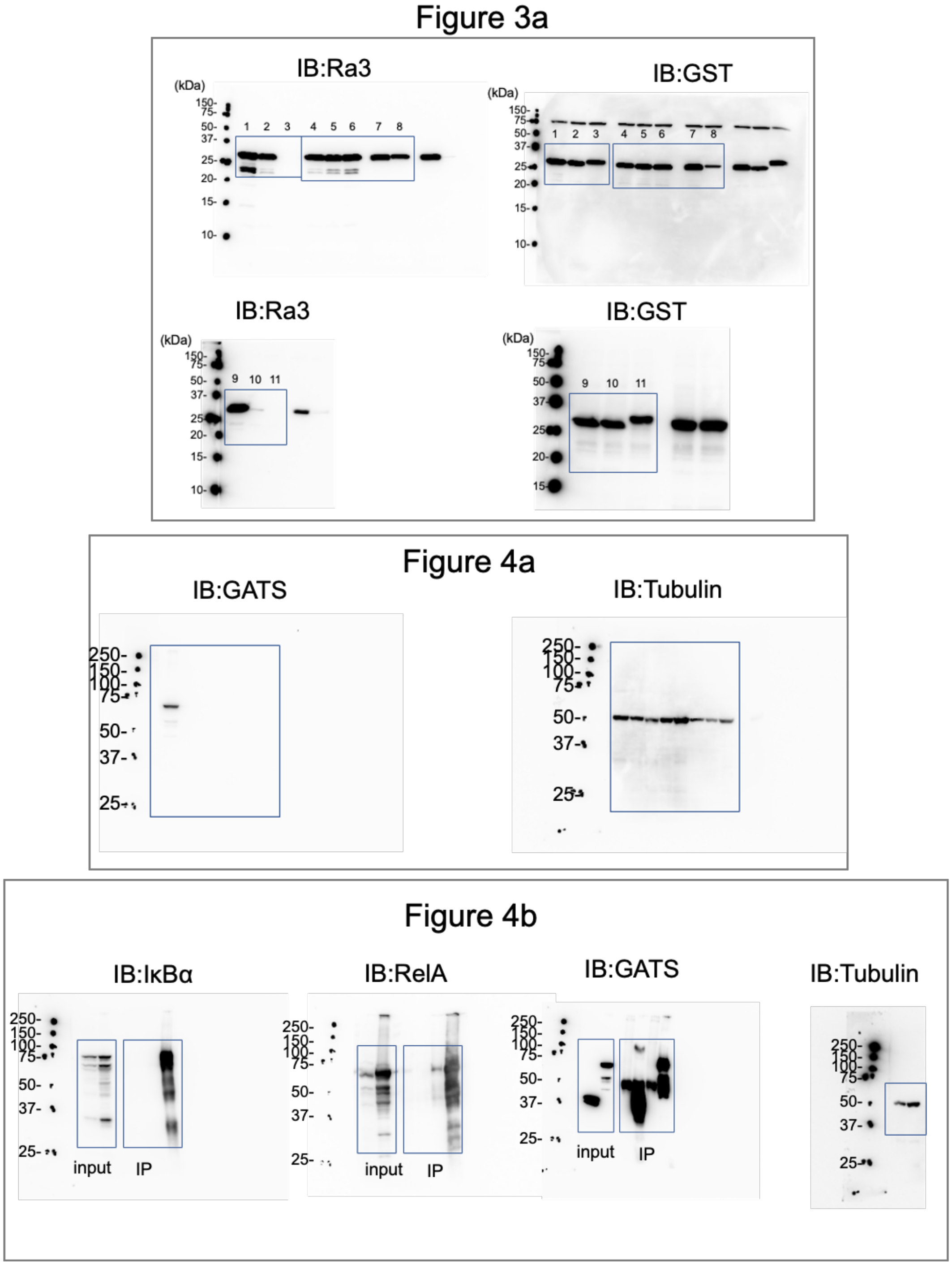

Supplementary Fig. 5 Full blot images of immunoblotting (Fig. 3-4)

All blots were performed using ImageJ.

Fig 5a

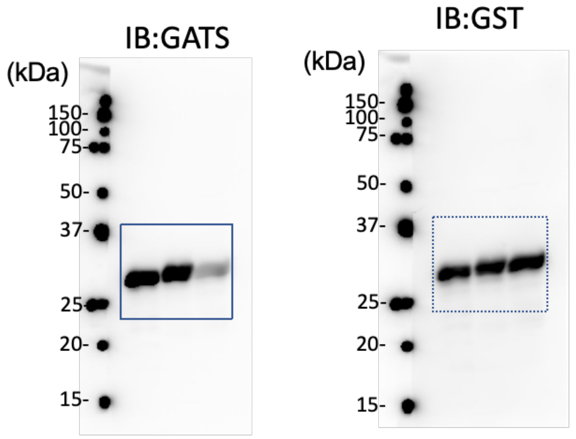

Fig 5b

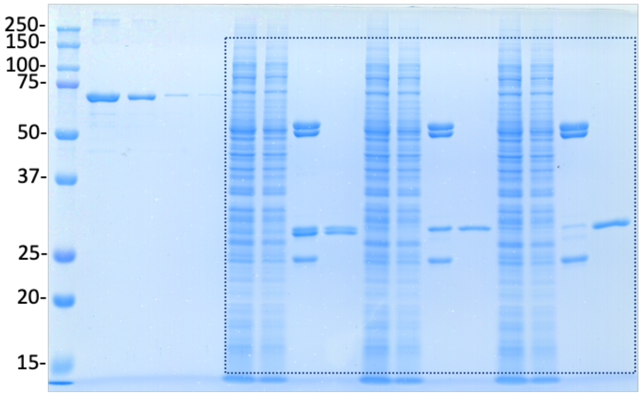

Fig 5c

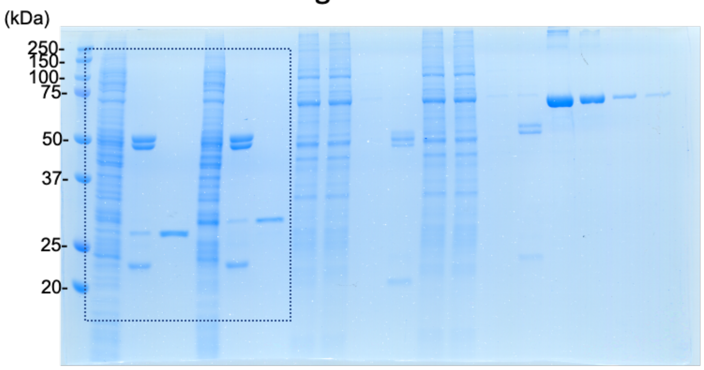

Supplementary Fig. 6 Full blot images of immunoblotting (Fig. 5)

All blots were performed using ImageJ.

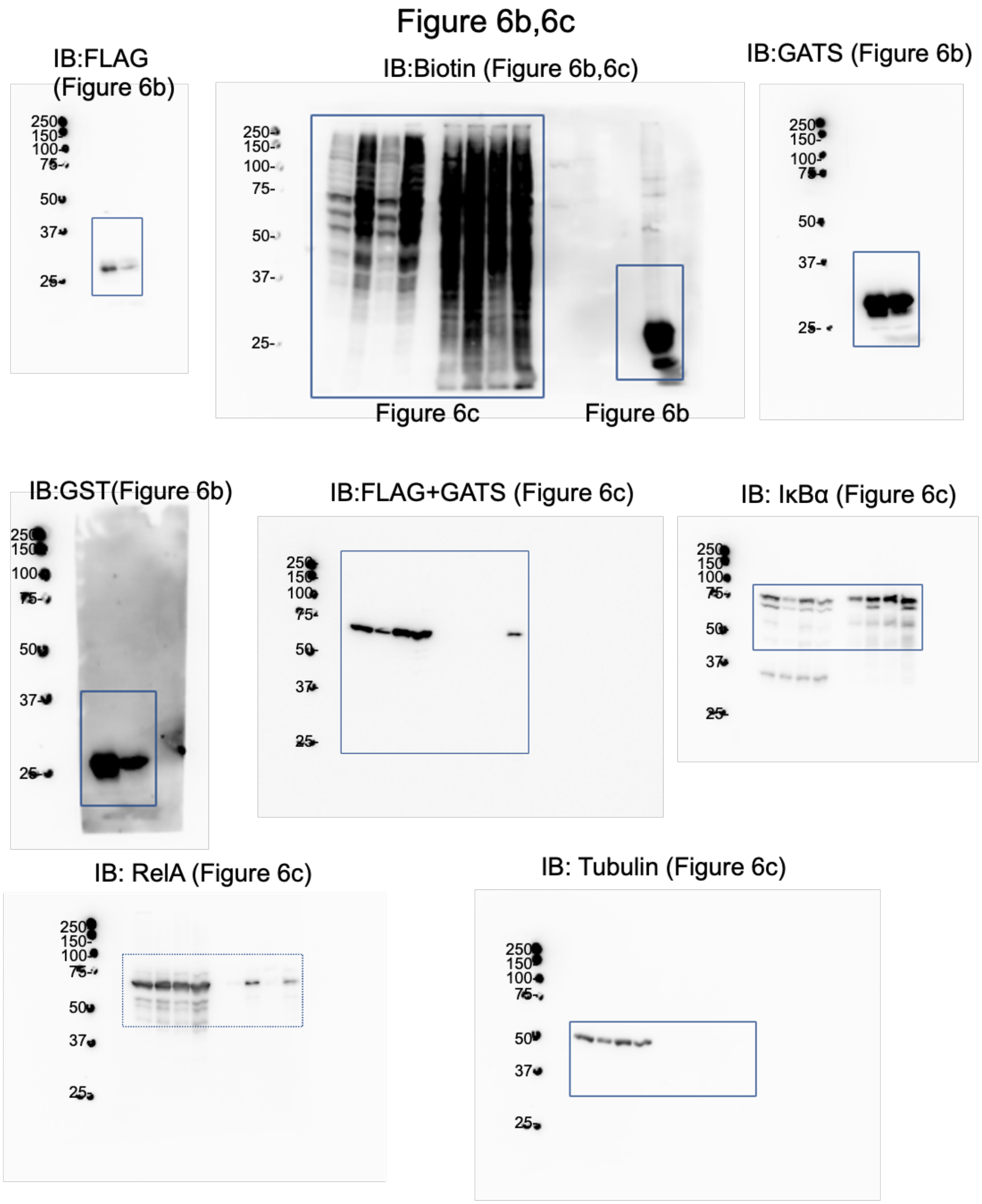

Supplementary Fig. 7 Full blot images of immunoblotting (Fig. 6)

All blots were performed using ImageJ.

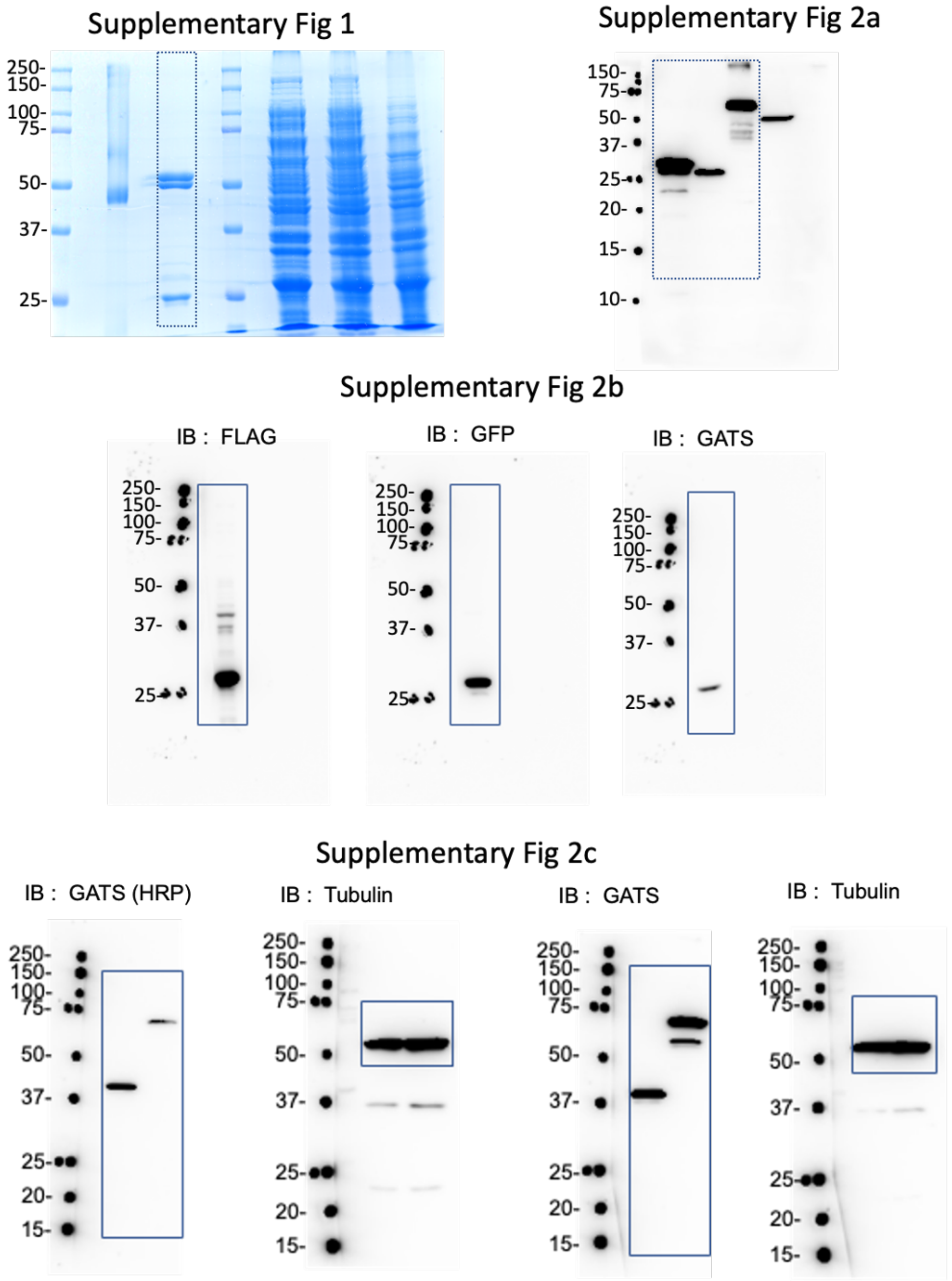

Supplementary Fig. 8 Full blot images of immunoblotting (Supplementary Fig. 1-2)

All blots were performed using ImageJ.

Supplementary Fig.3

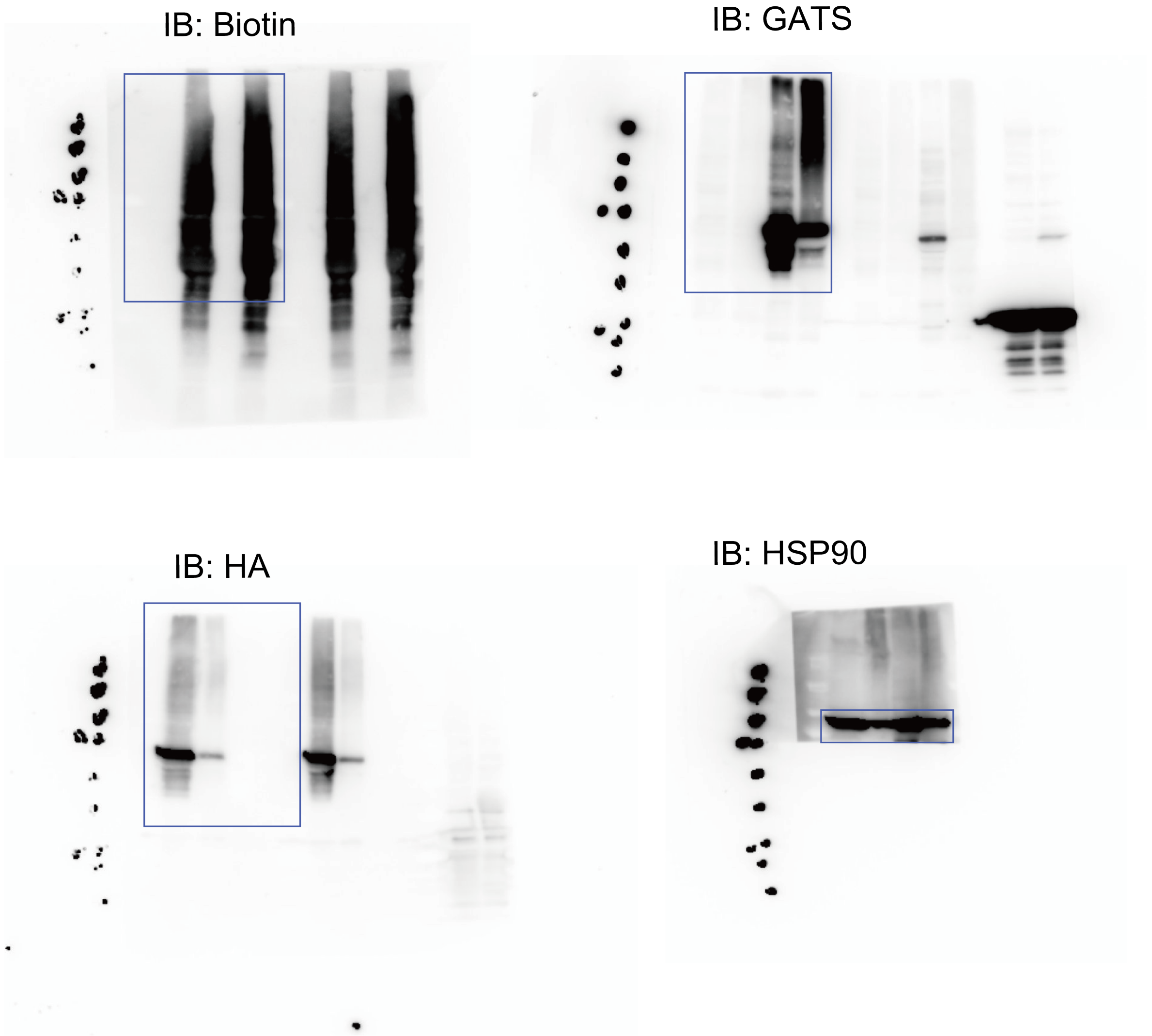

Supplementary Fig. 9 Full blot images of immunoblotting (Supplementary Fig. 3)

All blots were performed using ImageJ.
